# Associations of Vision Impairment with Cognitive Impairment Among Stroke Survivors in the Atherosclerosis Risk in Communities Study

**DOI:** 10.1002/alz.094600

**Published:** 2025-01-09

**Authors:** Kimberly Hreha, Marissa Ashner, Sarah Peskoe, Brian Downer, Timothy Reistetter, Heather Whitson

**Affiliations:** ^1^ Duke University, Durham, NC USA; ^2^ University of Texas Medical Branch, Galveston, TX USA; ^3^ UT Health San Antonio, San Antonio, TX USA

## Abstract

**Background:**

Vision impairment is a common complication of stroke, which has been linked to cognitive decline in stroke survivors. Little is known about how specific types of vision impairment influence the relationship between stroke and dementia. The aims of this project were: (1) to characterize the prevalence of both pre‐stroke and stroke‐related vision impairment(s) among stroke survivors with and without mild cognitive impairment (MCI) or dementia, and (2) to determine associations of vision impairment with cognitive impairment, depression, and physical performance.

**Methods:**

Data from the Atherosclerosis Risk in Communities Study (ARIC) were studied. Our analytic cohort included ARIC participants who had both a stroke hospitalization captured via surveillance prior to dementia and cognitive measures from Visits 5‐6. Participants without a retinal exam prior to their stroke were excluded. We determined the prevalence of specific types of vision impairments and ocular conditions as well as patient‐level demographics, stratified by cognitive status. Multivariable linear and logistic regressions, controlling for age, sex, and education, modelled the relationship between types of vision impairment and each outcome.

**Results:**

Our final cohort contained n = 227 individuals, with mean (standard deviation [SD]) age of 78.5 (5.56) years. Participants were primarily male (54.2%) and white (73.6%), with a mean of 14.5 (SD = 4.24) years of education. 15.9% had a pre‐existing vision impairment (e.g., glaucoma), and 18.5% developed a stroke‐related vision impairment (e.g., diplopia). Vision impairment (all types) was significantly associated with dementia (adjusted odds ratio (OR) = 2.17, 95% confidence interval (CI) = 1.07‐4.41), but not MCI (OR = 1.23, 95% CI = 0.07‐2.20). In assessing pre‐stroke vision impairment and stroke‐related vision impairment separately, there were no statistically significant associations with any outcomes.

**Conclusions:**

This study informs the rates of types of well‐characterized vision impairments in a group of stroke survivors. There is a 2‐fold increase in the likelihood of dementia among stroke survivors with vision impairment as compared to those without vision impairment, defined as either pre‐stroke or stroke‐related impairment. Future data collection in larger, more diverse populations is needed to determine how specific types of vision impairments in stroke survivors associate with cognitive impairment as well the likelihood of depression or worse functional status.

